# Vasa vasorum of proximal cerebral arteries after dural crossing — potential imaging confounder in diagnosing intracranial vasculitis in elderly subjects on black-blood MRI

**DOI:** 10.1007/s00330-021-08181-5

**Published:** 2021-08-04

**Authors:** Konstanze Viktoria Guggenberger, Giulia Dalla Torre, Ute Ludwig, Patrick Vogel, Andreas Max Weng, Marius Lothar Vogt, Matthias Fröhlich, Marc Schmalzing, Esther Raithel, Christoph Forman, Horst Urbach, Stephan Meckel, Thorsten Alexander Bley

**Affiliations:** 1 Department of Diagnostic and Interventional Radiology, University Hospital Wuerzburg, Faculty of Medicine, University of Wuerzburg, Oberduerrbacher Straße 6, 97080 Wuerzburg, Germany; 2 Department of Radiology, Medical Physics, Medical Center – University of Freiburg, Faculty of Medicine, University of Freiburg, Freiburg, Germany; 3 Department of Experimental Physics 5 (Biophysics), University of Wuerzburg, 97074 Wuerzburg, Germany; 4 Department of Diagnostic and Interventional Neuroradiology, University Hospital Wuerzburg, Wuerzburg, Germany; 5 Department of Internal Medicine II, Rheumatology and Clinical Immunology, University Hospital Wuerzburg, Wuerzburg, Germany; 6 Siemens Healthineers AG, Erlangen, Germany; 7 Department of Neuroradiology, Medical Center – University of Freiburg, Faculty of Medicine, University of Freiburg, Breisacher Straße 64, 79106 Freiburg, Germany

**Keywords:** Magnetic resonance imaging, Vasa vasorum, Large artery vasculitis, Atherosclerosis, intracranial arteries, Vertebral artery

## Abstract

**Objectives:**

Vessel wall enhancement (VWE) may be commonly seen on MRI images of asymptomatic subjects. This study aimed to characterize the VWE of the proximal internal carotid (ICA) and vertebral arteries (VA) in a non-vasculitic elderly patient cohort.

**Methods:**

Cranial MRI scans at 3 Tesla were performed in 43 patients (aged ≥ 50 years) with known malignancy for exclusion of cerebral metastases. For vessel wall imaging (VWI), a high-resolution compressed-sensing black-blood 3D T1-weighted fast (turbo) spin echo sequence (T1 CS-SPACE prototype) was applied post gadolinium with an isotropic resolution of 0.55 mm. Bilateral proximal intradural ICA and VA segments were evaluated for presence, morphology, and longitudinal extension of VWE.

**Results:**

Concentric VWE of the proximal intradural ICA was found in 13 (30%) patients, and of the proximal intradural VA in 39 (91%) patients. Mean longitudinal extension of VWE after dural entry was 13 mm in the VA and 2 mm in the ICA. In 14 of 39 patients (36%) with proximal intradural VWE, morphology of VWE was suggestive of the mere presence of vasa vasorum. In 25 patients (64 %), morphology indicated atherosclerotic lesions in addition to vasa vasorum.

**Conclusions:**

Vasa vasorum may account for concentric VWE within the proximal 2 mm of the ICA and 13 mm of the VA after dural entry in elderly subjects. Concentric VWE in these locations should not be confused with large artery vasculitis. Distal to these segments, VWE may be more likely related to pathologic conditions such as vasculitis.

**Key Points:**

• *Vasa vasorum may account for concentric VWE within the proximal 2 mm of the ICA and 13 mm of the VA after dural entry in non-vasculitic elderly people.*

• *Concentric enhancement within the proximal 2 mm of the intradural ICA and within the proximal 13 mm of the intradural VA portions should not be misinterpreted as vasculitis.*

• *Distal of this, VWE is likely related to pathologic conditions, in case of concentric VWE suggestive of vasculitis.*

## Introduction

Vasa vasorum is a term referring to typically microvascular structures or rather vessel networks, consisting of arteries, capillaries, and veins, physiologically occurring in the walls of larger extracranial arteries and veins, supplying them with oxygenated blood and nutrients and removing metabolic substances [1]. Both intra- and extracranial arteries are composed of three layers: the intima, media, and adventitia. Nutrition of the inner portions of the vessel walls is mainly ensured by diffusion from the luminal blood flow. Outer parts of extracranial vessel walls, however, do not benefit from the diffusing nutrients in the bloodstream, as they are out of reach. Therefore, these outer portions require additional supply with nutrients by vasa vasorum [2, 3]. Beyond, vasa vasorum also play a role in vascular pathology by favoring hypertrophy of the intima-media complex, atherosclerotic vessel wall changes, plaque hemorrhage, and dissection via rupture [4].

Vasa vasorum of intracranial arteries, in contrast, are rare, mainly due to the special anatomic construction and surrounding of intracranial arteries [2, 5, 6]. Nourishment of the outer parts of intracranial arteries is probably partly ensured by the nutrient-rich cerebrospinal fluid typically surrounding their adventitial surface [5]. Diffusion of nutrients from the luminal blood flow and the surrounding cerebrospinal fluid through the vessel wall is facilitated in intracranial arteries compared to extracranial arteries of comparable size due to their thinner media and adventitia [6]. Intracranial vasa vasorum usually develop with age, and proliferation is favorably influenced by different vasculopathies, particularly atherosclerosis [2], but also by inflammatory changes such as vasculitis. Intracranial vasa vasorum occur predominantly along the proximal intracranial vessel segments [7], shortly after the vessels’ dural crossing and usually fading in the further distal vessel course. In vessel wall imaging (VWI) studies using black-blood MRI, vasa vasorum typically cause a concentric mural contrast-enhancement and slight vessel wall thickening [8]. This type of vessel wall enhancement (VWE) and slight wall thickening may easily be mistaken for otherwise characteristic findings of inflammation related to cerebral vasculitis [9]. Primary angiitis of the central nervous system typically affects younger patients with no prior history of cardiovascular diseases with a mean age of the initial manifestation at the end of the fourth decade of life [10]. In this patient population, no relevant VWE caused by vasa vasorum is to be expected. Secondary central nervous system vasculitis, however, usually occurs in the framework of any other underlying pathology, often in the presence of other autoimmune diseases such as systemic forms of vasculitis, for example giant cell arteritis. The typical patient population with suspected giant cell arteritis and potential involvement of the intracranial arteries usually includes elderly patients, often with pre-existing vascular conditions. Therefore, concentric VWE of the proximal intracranial arteries related to the presence of vasa vasorum may be considered as a possible and frequent confounder of large artery vasculitis in the VWI assessment of intracranial arteries, potentially causing false positive results [9].

We hypothesized that concentric VWE may be commonly found on MRI VWI studies in elderly subjects. Thus, our aim was to determine the frequency and longitudinal extension of concentric VWE within the proximal cerebral arteries in a non-vasculitic cohort of elderly subjects, which may most likely relate to vasa vasorum. We used high-resolution 3D black-blood VWI with modern compressed-sensing (CS) technology for analysis of VWE in the proximal intradural portions of the internal carotid (ICA) and vertebral arteries (VA) after their dural crossings in order to build a reference base for future comparison with patients suspected of intracranial vasculitis.

## Methods

### Patient population

Following IRB approval for the study and after written informed consent, 43 consecutive patients with the following criteria were included in the study within 3 months: (1) age ≥ 50 years (mean age 71 years, standard deviation (SD) 10 years); (2) scheduled for contrast-enhanced cerebral MRI scan to rule out intracranial tumor manifestation (known cancer diagnosis); (3) all patients were clinically and laboratory asymptomatic regarding intracranial vasculitis and underwent contrast-enhanced cranial MRI as part of routine tumor staging.

The presence of intracranial metastases constituted an exclusion criterion.

### Imaging protocol

MRI was performed on a 3-Tesla scanner (MAGNETOM Prisma, Siemens Healthineers). In addition to standard MRI sequences, a dedicated sagittal compressed-sensing (CS)-accelerated, high-resolution black-blood 3D T1-weighted sampling perfection with application-optimized contrasts using different flip angle evolution (SPACE-) sequence (T1 CS-SPACE prototype) optimized for intracranial VWI as described elsewhere [11] was acquired in all patients approximately 5 min after injection of a gadolinium-containing contrast agent (Dotagraf®, 0.5 mmol/ml). The sequence provides a whole-brain coverage with an isotropic resolution of 0.55 mm within a scan time of 5:50 min by applying a CS-sampling factor of 0.22. A dedicated 64-channel head coil was used. Further scan parameters are as follows: TR/TE = 800/10 ms, FOV = 210 × 210 × 140 mm^3^, matrix = 384 × 384 × 256 px^3^, pixel-bandwidth = 450 Hz/px. Images were reformatted in axial and coronal sections.

### Image analysis

Images were analyzed by two readers independently. Both readers underwent specific training in interpreting vessel wall imaging including side-by-side analysis of 50 vessel wall MRI studies together with a senior board-approved radiologist and a senior board-approved neuroradiologist, both experts with 15 and 7 years of experience in reading vessel wall imaging studies, respectively.

Reader (R) 1 was a radiology resident with 2 years of dedicated neuroradiology training experience, and reader 2 was a radiology resident with 1 year of diagnostic radiology training. Both readers individually evaluated both ICAs and both VAs for the presence of VWE along the intracranial vessel course after the dural crossings. For the purpose of describing the anatomic localization of vessel wall changes, the internal carotid artery was subdivided into 7 segments according to the classification of Bouthillier et al [12], the vertebral artery was subdivided into 5 segments according to the angiographic classification [13]. Measurements were performed using the original 3D data sets after individually reformatting and adjusting to the respective vessel. Concentric-circumferential VWE (defined as affecting 360° of the vessel diameter) of the specific vessel in the proximal intracranial vessel course distal to the dural crossing was attributed to the presence of vasa vasorum. Eccentric VWE additionally to underlying concentric VWE was interpreted as combination of underlying enhancement caused by vasa vasorum and additional atherosclerotic wall lesion. The length of the affected vessel segment with concentric VWE until the point of fading was visually assessed and measured with an electronic caliper. Significantly discordant results were re-analyzed by both readers for consensus. In some cases, reliable measuring of the respective vessel segment was complicated by the tortuous vessel course of the proximal intracranial artery segments, despite the possibility for multiplanar reformatting of the 3D MRI data sets. For consensus reading of discordant results and for better assessment of these selected cases, we applied a dedicated post-processing tool that allows straightening of the respective vessel structure. Evaluation of the length of the enhancing vessel segment with the post-processing tool takes slightly longer time than the mere visual assessment and did not reveal to be more precise in the case of straight vessel segments. Therefore, the post-processing tool was used only in case of significant tortuosity of the respective vessels or in case of relevant discrepancy between the measurements of readers 1 and 2.

### Post-processing tool

The visualization of tortuous anatomies of arteries may not be applicable with single plane orientation. Therefore, a dedicated custom-built software framework was used to extract the desired structure in a 3D data set and prepared for planar visualization by extraction of the volume and identification of the vessel’s center line as reported elsewhere in [14] and in Guggenberger et al (under review in MAGMA). This resulted in a planar visualization of the cross-section through the vessel. The framework was able to extract structures directly from black-blood contrast MR data sets, which avoided running additional MRA sequences.

**Fig. 1:**
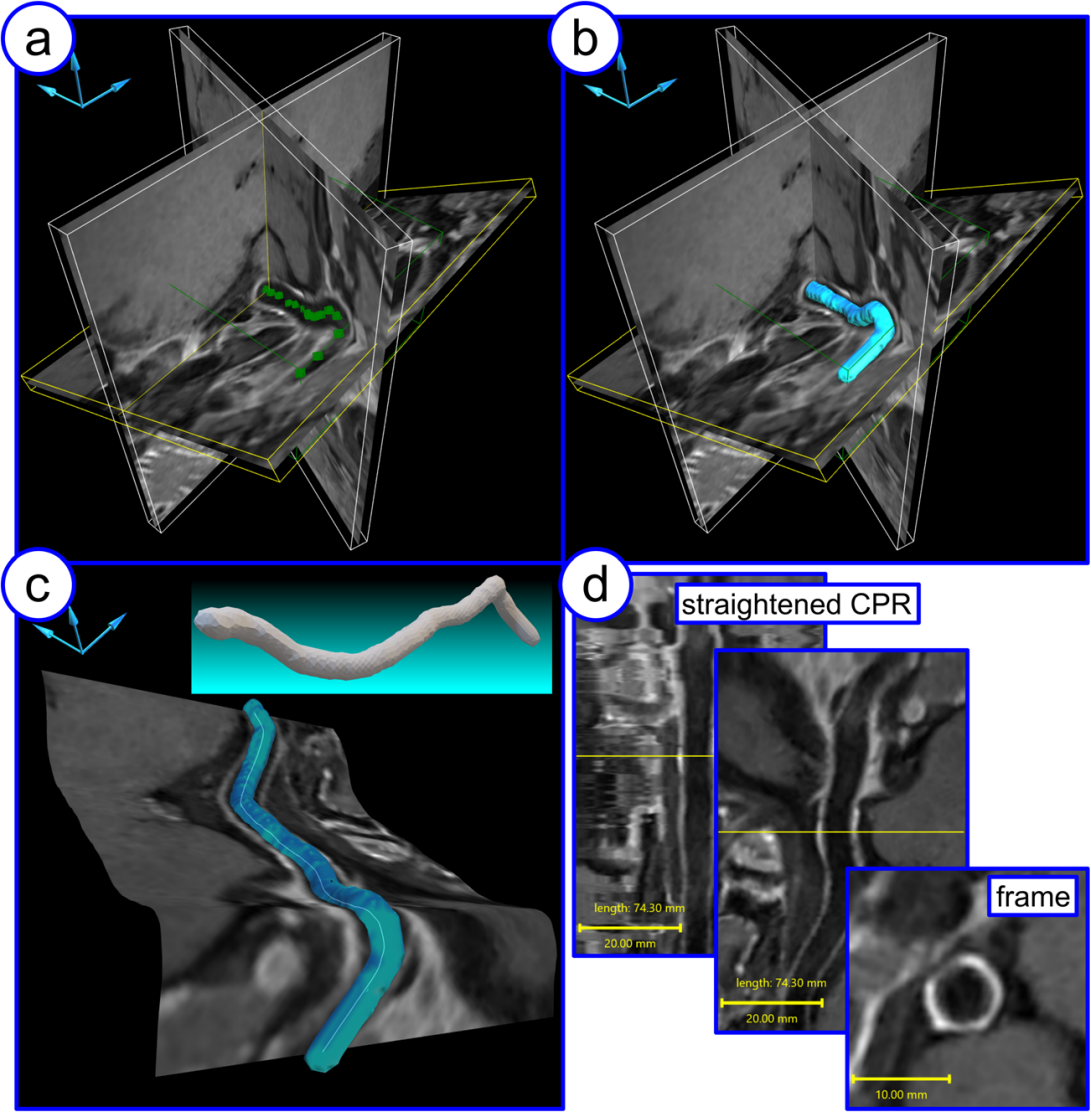
Step-by-step visualization process: After indicating manually the desired vessel structure (**a**), the inner lumen is extracted, and the surface was generated automatically (**b**). Based on these data, the centerline through the vessel structure was calculated (**c**) and the curved planar reformation (CPR) views for further assessment are provided (**d**).

The framework consisted of a 3D graphical user interface and was optimized for user-friendly and fast data processing with only a few semi-automated steps: in a first step, the desired area within the 3D data set was selected and restricted (Fig. 1a). With an automated step, the desired vessel structure was extracted [15], and the vessel surface was calculated (Fig. 1b) [16]. After extracting the voxel-wise centerline [17], a correction algorithm was applied, considering the surface of the vessel, to calculate a smooth 3D path through the center of the vessel (Fig. 1c) (reported in Vogel et al, under review in IEEE Transactions on Visualization and Computer Graphics). Utilizing the 3D path, perpendicular frames [18] along the structure were determined, which were used for calculation of reformatted views, the curved planar reformation (Fig. 1d) [19].

### Statistical analysis

Assuming normal distribution of the results, mean, range, and standard deviation were evaluated for descriptive statistics. A boxplot graph was used to visualize the distribution of measurements of both readers for each vessel. Bland-Altman plots were used to compare the length measurements of the enhancing vessel segments between both readers. Cronbach’s alpha was calculated for determining interrater reliability. Cronbach’s alpha ≥ 0.9 is considered excellent, ≥ 0.8 good, ≥ 0.7 acceptable, ≥ 0.6 questionable, ≥ 0.5 poor, and ≤ 0.5 unacceptable [20].

## Results

**Fig. 2:**
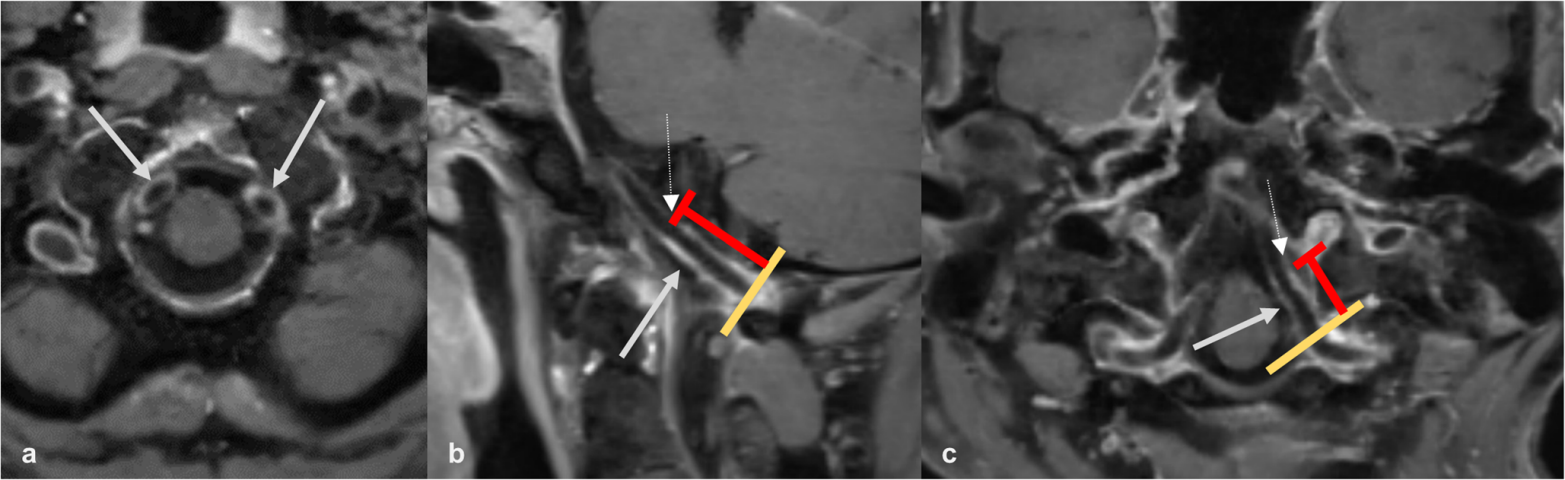
Multiplanar reconstructed images of post-contrast 3D CS T1 SPACE sequence show significant concentric VWE within proximal intracranial V4 segments of bilateral VAs in an asymptomatic patient likely related to vasa vasorum (**a–c**, white arrows). The red lines mark the length of the proximal intracranial VWE within the affected arteries, the dashed arrows mark the crossover to the normal, non-enhancing vessel wall (**b**, **c**). The yellow line marks the vessel’s dural crossing.

**Fig. 3:**
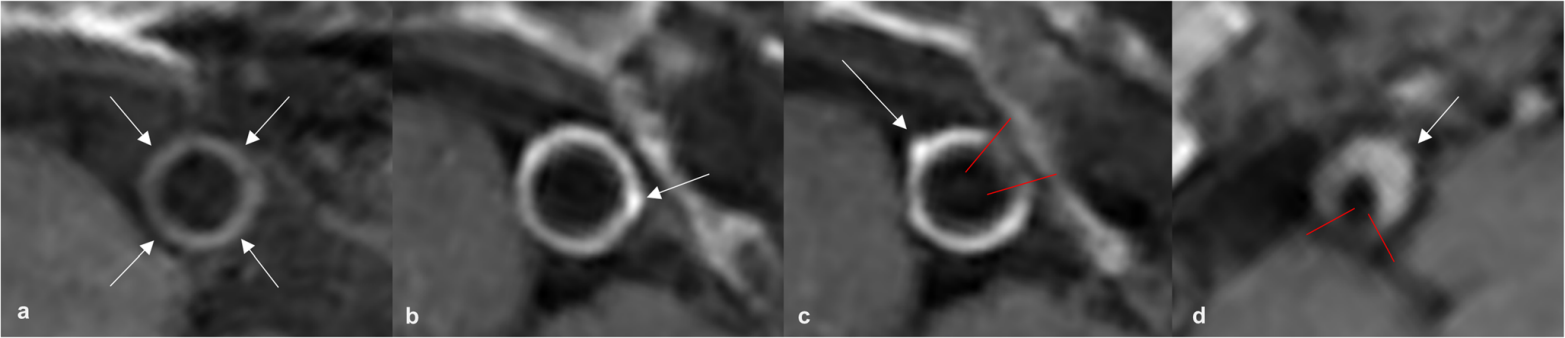
Axial reformats of the post-contrast 3D CS T1 SPACE VWI sequence with different types of vessel wall changes in three asymptomatic patients: Patient 1 (panel **a**) shows only typical mild and circumferent mural hyperintensity without relevant VWE of the proximal intracranial AV (**a**, white arrows). Patient 2 (panels **b** and **c**) exhibits substantial concentric VWE of the VA in the proximal V4 segment (**b**), additionally discrete single vessel wall irregularities (**b**, white arrow), interpreted as VWE caused by vasa vasorum with additional atherosclerotic wall lesions. Few millimeters further distal, the VA in the same. patient shows incomplete asymmetric VWE of the vessel’s circumference (**c**, red lines mark the enhancement’s borders) while wall irregularities, especially along the outer circumference of the vessel wall, facing away from the luminal blood flow, increase (**c**, white arrow). These findings may be interpreted as increasing atherosclerotic wall changes and fading vasa vasorum due to the incomplete coverage of the vessel wall circumference. Patient 3 (panel **d**) shows eccentric vessel wall thickening and VWE involving about 270° of the vessel wall’s circumference, interpreted as advanced atherosclerotic wall changes with outward remodeling due to the eccentric plaque-like configuration (**d**, white arrow, red lines mark the enhancement’s borders).

The main results are summarized in Table 1. Sole VWE of the ICA was never observed. Patients with VWE of the ICA always showed coexisting VWE of both VAs. In 13 of 39 cases (33%), the V5 segment of the vertebral artery was affected of at least one side. In 26 of 39 cases (66%), the VWE of the VA was limited to the V4 segment. The length of VWE of the proximal intradural artery segments was significantly longer in both VAs compared to the ICAs (see below Fig. 6). In 14 of 39 patients (36%) with proximal intradural VWE, the character of contrast-enhancement was smooth, delicate, and concentric likely related to the presence of vasa vasorum. In the remaining 25 patients (64 %), irregular thickening and mural enhancement of the vessel walls with eccentric plaques was disclosed, so that additional atherosclerotic lesions were assumed. Four of 43 (9 %) patients did not show any VWE at all (Figs. 2 and 3).

**Table 1:** Results for the mean length of vessel wall enhancement of the intradural VA and ICA.

	Total number (n) of patients	43 (27 male, 16 female)
VA	n (%) of patients with VWE of intradural vessel segments V4–V5	39 (91%), 37 bilateral
	Mean length of VWE in intradural vessel segments (mm)	13 (right VA: 12, left VA: 14) +/− 9 (SD); Overall range: 0–52 (right VA: 0–34, left VA: 0–52)
ICA	n (%) of patients with VWE of intradural vessel segments C6–C7	13 (30%), 11 bilateral
	Mean length of VWE in intradural vessel segments (mm)	2 (right ICA: 2, left ICA: 2) +/− 4 (SD); Overall range: 0–14 (right ICA: 0–13, left ICA: 0–14)

**Fig. 4:**
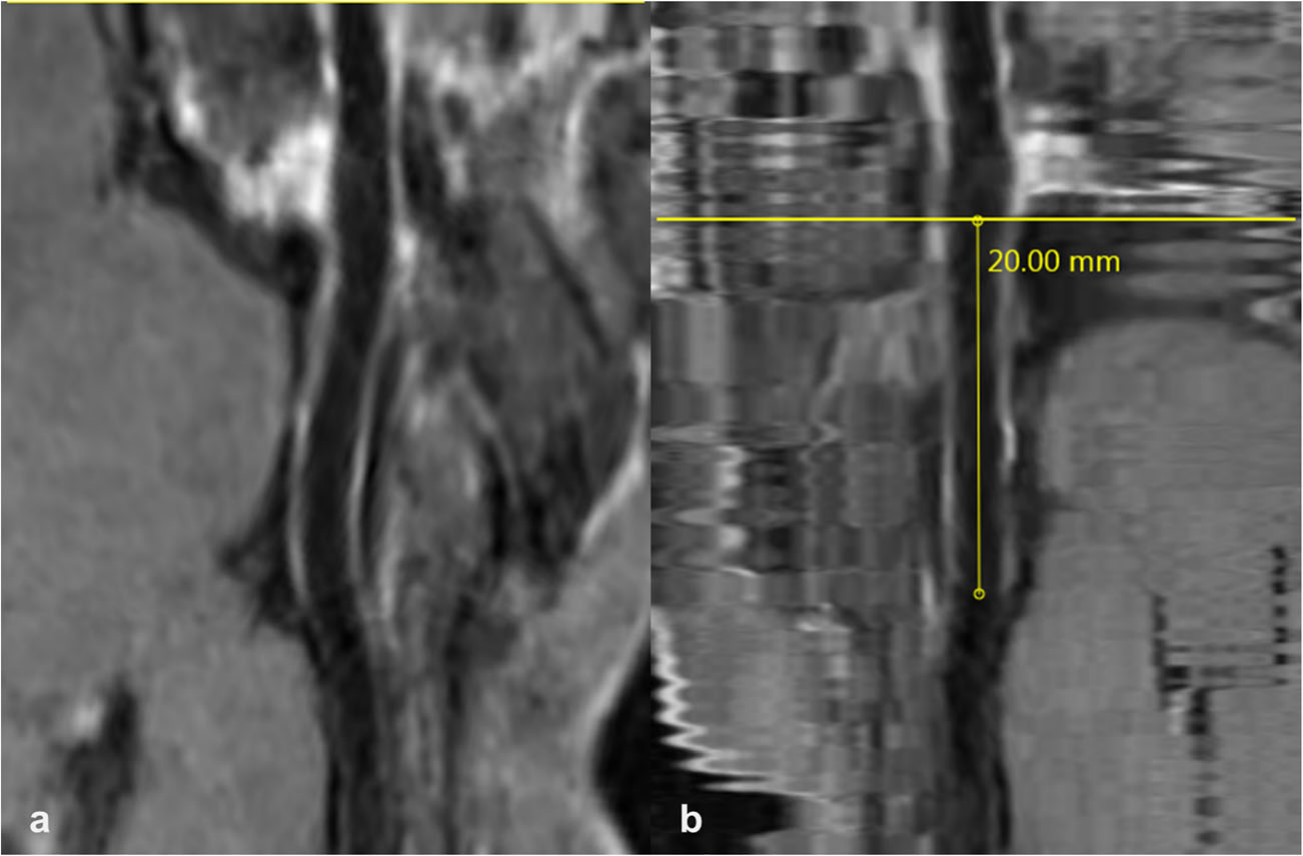
Post-contrast VWI of the tortuous course of the VA following preparation with the described reconstruction algorithm, depicted in the stretched (**a**) and straightened (**b**) form, the latter suitable for measurement. The yellow line marks the vessel’s dural crossing.

In 7 out of 43 patients (16 %), the results of assessment by readers 1 and 2 were significantly discordant due to a long-segmental VWE and a tortuous vessel course in the respective proximal intradural vessel segment. Reliable measurability of the curvy enhancing vessel segment is only possible to a limited extent with conventional methods. Therefore, these cases were re-analyzed after post-processing using a dedicated software algorithm allowing straightening of the respective vessel structures (Fig. 4).

### Statistical analysis

**Fig. 5:**
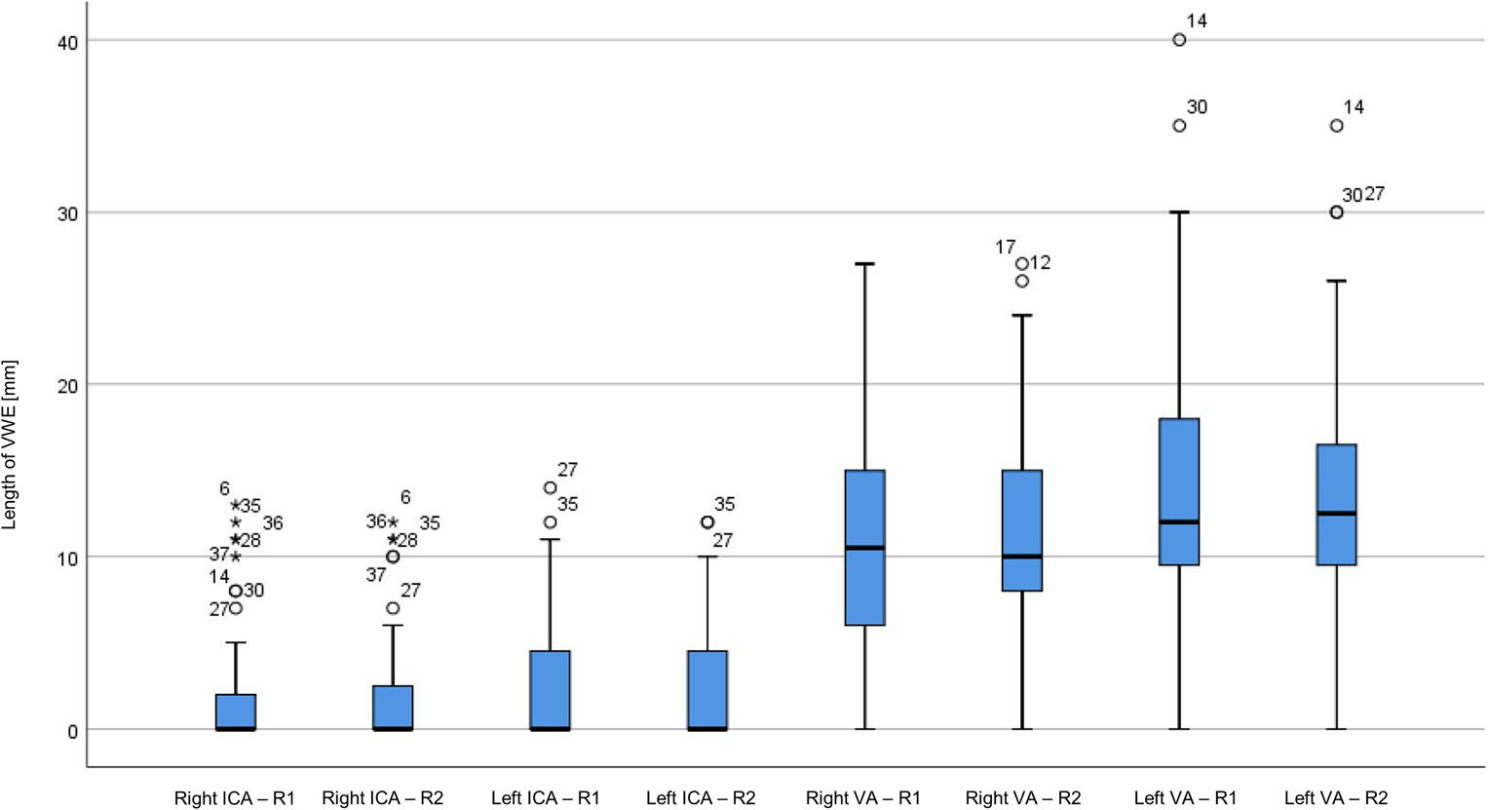
Boxplot graph showing that the range of distribution of measurements is comparable for both readers for the respective vessel with few outlier results for most of the analyzed vessels.

**Fig. 6:**
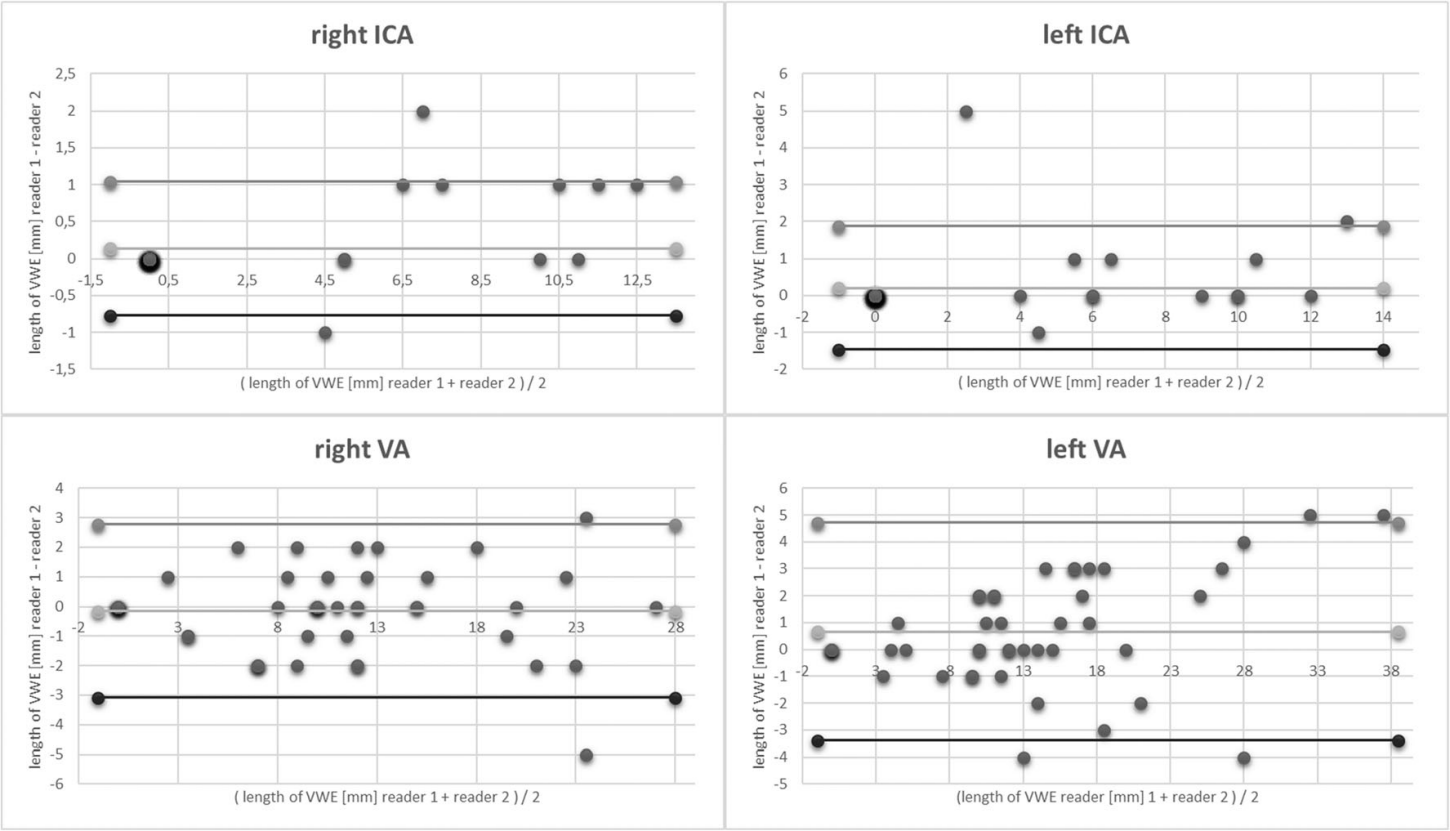
Bland-Altman plots revealing that most measurements of both readers lie within the standard deviation with few outlier values. In the case of the left VA, deviation from mean of VWE is getting larger with increasing length of the enhancing vessel segment. Increasing length of the proximal intradural artery segments most often comes along with increasing tortuosity of the respective vessel segment. This makes reliable and reproducible measurement difficult and emphasizes the importance of a mechanism for straightening intracranial vessel structures in order to enable reliable evaluability.

Cronbach’s alpha of 0.996 for the right ICA, 0.988 for the left ICA, 0.989 for the right VA, and 0.985 for the left VA indicate an excellent interrater agreement for the length of VWE (Figs. 5 and 6).

## Discussion

Concentric VWE within the proximal 2 mm of the ICA and within the proximal 13 mm of the VA after dural entry was detected in a substantial number (30 % in the ICA, and 91 % in the VA) of non-vasculitic elderly patients (aged ≥ 50 years) with extracranial malignant disease. This type of VWE is likely related due to vasa vasorum enhancement of the proximal intradural portions of the large cerebral arteries and should not be confused with VWE related to inflammation in intracranial vasculitis [2, 7, 9].

Diagnosing cerebral vasculitis using MRI VWI remains a challenge despite substantial technical progress: on the one hand due to the special anatomic conditions of intracranial arteries, being very thin vessel structures with a mostly tortuous course, rarely fitting standard reconstructions, and on the other hand due to a number of confounders and differential diagnoses with similar imaging findings [2, 9]. One important confounder is the presence of vasa vasorum along intracranial arteries as it can mimic concentric VWE otherwise characteristic for vasculitic affection [2, 9]. Vasa vasorum–related VWE typically occurs in the proximal vessel segments of intracranial arteries, right after their dural crossing [2]. There are no reference values in the literature regarding the length of intradural extension of VWE of the ICA and VA caused by vasa vasorum or recommendations defining the distance from these arteries’ dural entry to the point where a concentric VWE may be considered as rather suggestive of vasculitis. However, a benchmark for the typical length of VWE of the proximal intracranial artery segments caused by vasa vasorum might help in the differentiation from inflammatory changes. In most cases, the VWE due to the vasa vasorum is clearly differentiable from the normal appearance of the intracranial arterial vessel wall that is depicted isointense or mildly hyperintense on T1w black-blood VWI sequences [2, 9]. Sometimes mural enhancement caused by vasa vasorum tapers in the further vessel course resulting in a very smooth transition between enhancing and non-enhancing vessel wall segments so that the exact length of the VWE caused by vasa vasorum is visually hardly definable, which may explain a significant inter-reader variability.

Particularly in the elderly patient population that often suffers from increased vascular comorbidities, there are frequently additional atherosclerotic vessel wall changes that might cause difficulties in the interpretation [2, 9]. Moreover, it can be assumed that the presence of vasa vasorum is associated with the progression of atherosclerosis [7, 21]. While vasculitic affection and VWE caused by the presence of vasa vasorum usually manifest as smooth concentric lesions, atherosclerotic vessel wall lesions typically show an eccentric plaque-like configuration [9]. Therefore, it is essential to assess any thickened and enhancing vessel segments in all three spatial dimensions in order to differentiate the underlying pathology [9]. However, in practice and especially in elderly patients, VWE and wall irregularities are often due to a combination of both, the presence of vasa vasorum and additional atherosclerotic vessel wall changes. In our cohort, VWE in 25 of 39 patients (64 %) showed an irregular, asymmetric, or eccentric configuration, supposedly related to a combination of vasa vasorum and atherosclerotic lesions.

Using black-blood VWI MRI, assessment of intracranial vessel wall structures with a mostly tortuous vessel course requires special technical features. Measurements were implemented on a dedicated sagittal 3D T1-weighted SPACE-sequence, optimized for intracranial VWI with ultra-high resolution due to the compressed-sensing technique as described previously [11]. In some cases, multiplanar reconstruction of high-resolution 3D VWI data is not sufficient to allow reliable measurement of the respective enhancing vessel segment. We used a dedicated post-processing tool enabling reconstruction and visualization of the vessel in stretched and straightened form, thus facilitating a precise measurement of the vessel segment in question.

Our study was not without limitations: First, the retrospective nature and the relatively small sample size makes it prone to potential selection bias. Thus, further studies with larger non-vasculitic cohorts are needed to support the results of this study. However, the analyzed patient cohort with an age ≥ 50 years represents an age-matched control group to the patients typically affected by giant cell arteritis with potential involvement of the cerebral arteries and with comparable atherosclerotic disease burden and associated vessel wall changes. The analyzed patients all had a history of extracranial malignancy not known to be associated with pathologic vessel wall changes (Table 2). Second, the length of vessel wall enhancement is difficult to be precisely measured in some cases due to the sometimes very smooth transition between enhancing and non-enhancing vessel wall segments and the oftentimes tortuous vessel course. Application of the dedicated post-processing tool facilitated length measurement of the vessel segment in question. Furthermore, meaningfulness of the study is limited due to the lack of histological proof which may be hard to obtain in patients with extracranial malignant disease but of otherwise good neurological health status. However, all studied patients neither revealed clinical signs or suspicion of vasculitis in history nor had laboratory signs of vasculitis at the time of the MRI scan, so that the vessel wall changes can be plausibly attributed to the presence of vasa vasorum and atherosclerotic changes

**Table 2:** Malignant diseases of the cohort with the respective number of affected subjects.

Malignant disease without intracranial manifestation	Number of patients
Melanoma	31
Bronchial carcinoma	7
Diffuse large B-cell lymphoma	2
Squamous cell carcinoma	1
Merkel cell carcinoma	1
Renal cell carcinoma	1

Previous histopathologic studies, however, have revealed results that make the imaging results of our study plausible: One histopathologic study revealed that vasa vasorum occur significantly more often in the proximal intracranial vessel segments than in the distal vessel segments, particularly in the VA and the ICA [7]. Another histopathologic study examined 30 human autopsy materials and found vasa vasorum in the proximal 1–2 cm of all VA and ICA [22], comparable to our imaging results. Another histopathologic study demonstrated that vasa vasorum were found in nearly half of all examined arteries with a significant higher prevalence in the VA compared to the basilar artery or the middle cerebral artery as well as a correlation between the existence and amount of vasa vasorum and the atherosclerotic disease burden in the VA [21].

## Conclusion

Concentric VWE within the proximal 2 mm of the intradural ICA and within the proximal 13 mm of the intradural VA portions was found in non-vasculitic elderly patients (aged ≥ 50 years). This finding presumably represents vasa vasorum–related VWE without pathological significance and should not be misinterpreted as intracranial vasculitis. Distal to these segments, VWE is more likely related to pathologic conditions such as vasculitis.

## Abbreviations

CS: Compressed-sensing
ICA: Internal carotid artery
MRI: Magnetic resonance imaging
R: Reader
SD: Standard deviation
SPACE: Sampling perfection with application-optimized contrasts using different flip angle evolution
VA: Vertebral artery
VWE: Vessel wall enhancement
VWI: Vessel wall imaging

## Declarations

### Guarantor

The scientific guarantor of this publication is Prof. Dr. Thorsten Alexander Bley.

### Statistics and Biometry

Dr. Andreas Max Weng (one of the co-authors) kindly provided statistical advice for this manuscript.

### Methodology

• prospective

• observational study

• multicenter study
